# The role of acute changes in mBDNF, cortisol and pro-BDNF in predicting cognitive performance in old age

**DOI:** 10.1038/s41598-023-35847-5

**Published:** 2023-06-09

**Authors:** Jonna Nilsson, Maria Ekblom, Marcus Moberg, Martin Lövdén

**Affiliations:** 1 Swedish School of Sport and Health Sciences, Stockholm, Sweden; 2 Aging Research Center, Karolinska Institutet and Stockholm University, Stockholm, Sweden; 3 Department of Neuroscience, Karolinska Institutet, Stockholm, Sweden; 4 Department of Psychology, University of Gothenburg, Gothenburg, Sweden

**Keywords:** Cognitive ageing, Cognitive neuroscience, Learning and memory, Neurotrophic factors

## Abstract

The interplay between biomarkers of relevance to neuroplasticity and its association with learning and cognitive ability in old age remains poorly understood. The present study investigated acute changes in plasma concentrations of mature brain-derived neurotrophic factor (mBDNF), its precursor protein (pro-BDNF), and cortisol, in response to acute physical exercise and cognitive training interventions, their covariation and role in predicting cognitive performance. Confirmatory results provided no support for mBDNF, pro-BDNF and cortisol co-varying over time, as the acute interventions unfolded, but did confirm a positive association between mBDNF and pro-BDNF at rest. The confirmatory results did not support the hypothesis that mBDNF change following physical exercise were counteracted by temporally coupled changes in cortisol or pro-BDNF, or by cortisol at rest, in its previously demonstrated faciliatory effect on cognitive training outcome. Exploratory results instead provided indications of a general and trait-like cognitive benefit of exhibiting greater mBDNF responsiveness to acute interventions when coupled with lesser cortisol responsiveness, greater pro-BDNF responsiveness, and lower cortisol at rest. As such, the results call for future work to test whether certain biomarker profiles are associated with preserved cognition in old age.

## Introduction

Mature brain-derived neurotrophic factor (mBDNF) is a neurotrophin that is essential for neuronal plasticity and learning^1–3^. It is produced as a precursor protein, pro-BDNF, which has the opposite biological function to mBDNF, negatively affecting neuronal plasticity when overexpressed^4,5^. Cortisol, which is a glucocorticoid involved in mediating the stress response, has also been shown to have negative effects on neuronal plasticity^6,7^. The apparently opposing effects of mBDNF and cortisol on neuroplastic processes has led to the working hypothesis that the two are part of an integrative system and that some of the negative effects of glucocorticoids on plasticity may be a consequence of attenuating mBDNF expression or signaling^8,9^. Aging is associated with declining neuroplasticity and cognition^10,11^, as well as with increasing cortisol levels and decreasing mBDNF levels^12,13^, implying that higher cortisol levels may attenuate mBDNF and thereby diminish neuroplasticity processes and cognition in old age. Similarly, the balance between mBDNF and pro-BDNF has been proposed to be important for the functional effects on neuroplasticity^14^. If mBDNF, cortisol and pro-BDNF are indeed functionally related, peripheral concentrations of such markers may be expected to co-vary over time, also in the acute term. An improved understanding of how biomarkers of relevance to neuroplasticity change and covary in response to acute interventions, as well as how their interactions predict cognitive performance in old age, is important from a mechanistic perspective and has the potential of contributing to the design of personalized interventions to improve the cognitive health of aging adults.

In the periphery, acute physical exercise leads to transient increases in both mBDNF concentrations^15–17^ and cortisol concentrations^18^, but the relationship between the two in this acute phase remains largely unknown^19,20^. Less is known regarding exercise-induced changes in pro-BDNF, with two human studies reporting no change in response to exercise or training in the periphery^21,22^. The relationship between acute changes in mBDNF and proBDNF is also largely unknown, but a positive relationship has been demonstrated between baseline levels of mBDNF and pro-BDNF in one study of older adults^23^.

Regarding the effect of cognitive engagement on peripheral concentrations of mBDNF, pro-BDNF and cortisol, next to nothing is known. To the extent that cognitive training reflects learning, biomarkers with relevance to neuroplastic processes, such as mBDNF, pro-BDNF and cortisol, may play a role^3,24^. It has previously been shown that mBDNF concentrations increase acutely in plasma following cognitive training in healthy older adults, but not in serum^25,26^. Regarding pro-BDNF and cortisol, the effect of cognitive engagement on peripheral concentrations is to our knowledge unknown.

The first aim of the present study was simply to describe the acute effects of physical exercise and cognitive training on pro-BDNF and cortisol concentrations in plasma, by expanding the biomarker analysis of an already completed randomized-controlled study in healthy older adults^25^. Whilst the effects of physical exercise and cognitive training on mBDNF has already been reported in a largely overlapping study sample^25,27^, descriptive data on mBDNF was nevertheless reported to allow for a qualitative comparison of patterns of change in all three biomarkers.

The second aim was to test a set of hypotheses pertaining to associations between rest levels and acute changes in mBDNF and cortisol, and between mBDNF and pro-BDNF, in the context of four acute interventions that involved either physical exercise prior to or following cognitive training, physical exercise only or cognitive training only. Following proposals that cortisol may attenuate the expression and signaling of mBDNF^8^, it was hypothesized that greater acute changes in cortisol would weaken parallel changes in mBDNF, resulting in a negative association, independent of intervention type (Hypothesis 1). Based on the same reasoning, it was hypothesized that cortisol at rest would be negatively related to acute changes in mBDNF (Hypothesis 2), as well as to mBDNF at rest (Hypothesis 3). Extending this to pro-BDNF, acute changes in pro-BDNF were hypothesized to be related to acute changes in mBDNF, independent of intervention type (Hypothesis 4). Based on previous findings, it was furthermore hypothesized that BDNF and pro-BDNF would be positively associated at rest (Hypothesis 5^23^).

The third and final aim of the study was to investigate whether acute changes in mBDNF, cortisol and pro-BDNF interact in predicting cognitive training outcome, which was an aim that followed directly from the primary finding of a randomized-controlled study in healthy older adults^25^. In this study, participants were randomized to complete a 12-week intervention, where each of the 30 intervention sessions comprised cognitive training preceded by physical exercise (PE + COG), cognitive training followed by physical exercise (COG + PE), physical exercise only (PE), or cognitive training only (COG). In the original study, we hypothesized that administering physical exercise immediately before cognitive training would mean that the transient exercise-induced mBDNF increase would coincide with and facilitate cognitive training outcome. Contrary to this, average cognitive training outcome did not differ between the groups that received physical exercise before or after cognitive training. However, acute increases of plasma mBDNF following physical exercise at pretest were associated with greater cognitive training gains in a restricted set of cognitive tasks, but only when cognitive training was preceded by physical exercise. We reasoned that any beneficial effect of the exercise-induced mBDNF increase on subsequent cognition may have been counteracted by other temporally coupled exercise-induced changes, preventing an effect to emerge at the group level. The last aim was therefore to test a set of hypotheses pertaining to whether temporally coupled exercise-induced changes in cortisol (Hypothesis 6) and pro-BDNF (Hypothesis 7), and resting cortisol levels (Hypothesis 8), counteracted an average beneficial effect of mBDNF on cognitive training outcome, when physical exercise preceded cognitive training. Hypotheses were pre-registered and are presented in full in the result section (https://osf.io/yuac9).

The original study was retrospectively registered as a clinical trial at ISRCTN (ISRCTN13543922, 18/09/2019).

## Methodology

### Participants

The original study was approved by the ethical review board in Stockholm (Regionala Etikprövningsnämnden, Stockholm, case number 2017/1115-31/4) and conducted in accordance with the Declaration of Helsinki^25^. The present study, which constituted an extended blood analysis of blood samples collected as part of the original study, was also approved by the ethical review board in Stockholm (Regionala Etikprövningsnämnden, Stockholm, case number 2018/329-32) and conducted in accordance with the Declaration of Helsinki. Out of the 97 participants who provided informed consent and completed the original study, 93 participants also provided informed consent for the present study. The present analyses were therefore limited to the 93 participants who had consented to participation in the original study as well as to the extended blood analyses of the present study.

All participants were between 65 and 75 years of age and cognitively unimpaired, as indicated by a MMSE score of 26 or higher. Participants were also physically healthy, defined as the absence of neurological disease, Parkinson’s disease or epilepsy, current or ongoing cardiovascular disease, history of brain damage, ongoing cancer, psychiatric illness and history of head trauma with resulting unconsciousness. Uncontrolled metabolic disease, including diabetes and Grave’s disease, also constituted an exclusion criterion. As such, participants with diabetes were included in the study, as long as their condition was adequately controlled via pharmacological intervention or lifestyle. Medications that could influence the blood analysis or fitness test, including antiepileptic and antidepressant medication, sleeping medication, cortisone treatment, beta blockers and beta-stimulants, were also exclusion criteria. For complete study criteria, please see the supplementary materials in the original publication^25^.

### Design and procedure

#### Blood sampling

**Figure 1:**
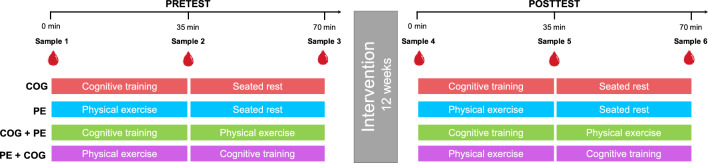
Blood sampling protocol. Acute changes in biomarkers concentrations were measured before and after the 12-week intervention period. Blood was drawn immediately before and after the first intervention, and immediately after the second intervention or after seated rest, at pretest (samples 1–3) and at posttest (samples 4–6). *COG* cognitive training only, *PE* physical exercise only, *COG + PE* cognitive training and then physical exercise, *PE + COG* physical exercise and then cognitive training.

The blood sampling protocol assessed acute changes in biomarker concentrations in response to the acute interventions at pretest and posttest (Fig. 1). An intravenous catheter was inserted into the antecubical vein upon arrival, and after 15 min of seated rest, the first sample was drawn. Given the absence of physical or cognitive engagement at this timepoint, the first blood sample was considered a baseline measure, reflecting biomarker concentrations at rest. Participants subsequently commenced with 35 min of cognitive training or 35 min of physical exercise, depending on the randomly allocated intervention, after which the second sample was immediately collected. Participants then engaged in 35 min cognitive training, 35 min physical exercise or 35 min seated rest, also depending on intervention, after which the third sample was drawn. Thus, a total of six blood samples were drawn from each participant, three at pretest (Sample 1–3) and three at posttest (Sample 4–6). To avoid contamination and clotting between samples, the catheter was flushed with saline solution between each draw. The blood sampling session was always scheduled for the first half of the day, starting at 7:45, 09:15 or 10:45, with the first blood sample being drawn at 08:00, 09:30 or 11:00. The timing was kept the same for each participant at pre-test and post-test and was counterbalanced so that each blood sampling session started at the same average time for the four intervention groups. Participants were instructed not to consume alcohol or engage in any physical training 24 h before blood sampling, and to not eat or drink anything 2 h before blood sampling. Participants were also required to record their breakfast at pretest and were instructed to have the same breakfast at posttest.

For each sample, 10 mL was collected into heparinized tubes. The blood was spun at 4 °C for 3 min at 6000 rpm to separate the plasma, which was transferred to Eppendorf tubes and frozen at − 80 °C until analysis. mBDNF concentrations were quantified with samples in duplicate using enzyme-linked immunosorbant assay (ELISA) according to the manufacturer’s instructions (Human BDNF Quantikine Immunoassay, DBD00, R & D Systems). Plasma levels of cortisol (ng × ml^−1^) were determined in duplicates using an ELISA Cortisol kit (#CO368S, Calbiotech, CA, USA). Plasma levels of proBDNF (pg × ml^−1^) were quantified using the Human Pro-BDNF DuoSet ELISA kit (DY3175) combined with the DuoSet Ancillary Reagent Kit 2 (DY008) from R&D Systems (MN, USA). Assays were run according to the manufacturer’s instructions with minor modifications and with samples analyzed in duplicates. The modifications included sample and standard incubation over night at 4 °C instead of two hours at room temperature, as well as standard dilution in 10% fetal bovine serum supplemented with 0.01% Tween-20 instead of the supplied diluent, which was done in order to reduce background signal in the assay.

#### Cognitive training outcome

In the original investigation, cognitive training outcome was assessed using 18 cognitive tests at pretest and posttest, selected to systematically vary in similarity to the cognitive training tasks, which targeted the updating construct of working memory^25^. In the original study, the hypothesized closer relationship between exercise-induced increases in mBDNF and cognitive training outcome when cognitive training was preceded, as opposed to followed by physical exercise, was found only for the cognitive composite that captured performance on trained updating working memory tasks with untrained stimuli. Since the hypotheses pertaining to cognitive training outcome in the present study built directly on this finding, the same trained working memory tasks were used as the measure of cognitive training outcome for hypothesis testing (H6–H8). As such, the used cognitive composite was based on one n-back task and one running span task, both of which targeted the updating construct of working memory and were part of the cognitive training program, but with a different set of stimuli. In the n-back task, stimuli were presented one by one and participants had to press a button whenever the stimulus was the same as the one presented N steps back in the series. In the running span task, stimuli were also presented one by one and when stimuli presentation stopped, participants had to select the last N stimuli from a set of options, in the right order. More detailed descriptions of all the cognitive tests can be found in the supplementary materials of the original publication^25^.

For exploratory purposes, specifically to test generalizability of results beyond the single cognitive composite used for hypothesis testing, the remaining tasks that targeted the updating construct of working memory were used, contributing to two additional cognitive composites. The first composite was based on the exact n-back and running span tasks that were part of the cognitive training program (with identical stimuli), and the second composite was based on a numerical updating task and a spatial updating task that was not part of the cognitive training. In the numerical updating task, four digits were presented briefly before they disappeared and participants were required to perform eight mathematical updating operations on the digits kept in working memory. In the spatial updating task, three 3 × 3 grids were presented. Three dots were presented briefly, one in each grid, before they disappeared and participants were required to perform spatial shifting operations on the locations kept in working memory.

### Statistical analysis

#### Biomarker concentrations

Concentrations in plasma were available for all 93 participants for all biomarkers. For mBDNF and pro-BDNF, concentrations were severely skewed at all sampling timepoints (skewness > 3.0, kurtosis > 10.0). For cortisol, concentrations were not normally distributed at the four first timepoints (skewness > 3.0 and/or kurtosis > 10.0). Natural log-transformations were therefore performed for all biomarker concentrations at all timepoints, which resulted in approximate normality for all measures (skewness < 3.0, kurtosis < 10.0). Extreme outliers were subsequently removed using the outlier labelling rule (IQR = 3.0), resulting in exclusion of one measurement for mBDNF, three for pro-BDNF, and one for cortisol. Repeated-measures ANOVA, with Time (pretest, posttest) and Sample (1–3) as within-subject factors and Intervention (COG, PE, COG + PE, PE + COG) as between-subject factor, was performed for each biomarker for descriptive purposes at the group level (Jamovi, version 1.6.23.0). Significant main effects were only reported and interpreted in the absence of significant interactions.

#### Area under the curve for biomarker change

For hypotheses 1–5, acute change in biomarker concentration over the three sampling timepoints, at pretest and at posttest, were expressed as the area under the curve with respect to change (AUC), as put forward by Pruessner, Kirschbaum^28^. As such, AUC were derived from the trapezoid formula in reference to the first value, ignoring the distance from zero for all timepoints, thereby emphasizing the changes over time. In contrast to change scores, AUC allows measurements from all three timepoints to be comprised in a single measure and was therefore selected for capturing change in biomarker concentration as the acute interventions unfolded. As such, AUC can be said to capture overall biomarker propensity to change in response to the acute interventions. AUC were calculated for mBDNF (mBDNF_AUC_), pro-BDNF (Pro-BDNF_AUC_) and cortisol (Cortisol_AUC_), at pretest and posttest, for participants in all four intervention conditions. All AUC variables were approximately normally distributed (skewness < 3.0, kurtosis < 10.0), without extreme outliers (outlier labelling rule, IQR = 3.0).

#### Change scores for biomarker change

For hypotheses 6–8, which built directly on our previous findings^25^, change scores were calculated to arrive at a measure of acute biomarker change following physical exercise in the groups that received both physical exercise and cognitive training (PE + COG, COG + PE). The concentration measured before exercise was subtracted from the concentration measured immediately after. For the group with exercise as their first intervention (PE + COG), sample 1 was therefore subtracted from sample 2, and for the group with exercise as their second intervention (COG + PE), sample 2 was subtracted from sample. 3. Change scores representing acute change following physical exercise were calculated for mBDNF (mBDNF_EX_), pro-BDNF (Pro-BDNF_EX_) and cortisol (Cortisol_EX_).

#### Cognitive composite

The cognitive composite used for hypothesis testing (trained tasks, untrained stimuli) was created by taking the unit-weighted average of the two tasks, at pretest and posttest, and composite scores were subsequently standardized by pretest performance ((x-mean_pretest_)/sd_pretest_) and converted into T scores (mean_pretest_ = 50, sd_pretest_ = 10). The same procedure was followed for the cognitive composites that were used for exploratory analyses. All three cognitive composite was approximately normally distributed at pretest and posttest (skewness < 3.0, kurtosis < 10.0).

#### Linear mixed-effect modelling

Linear mixed-effects models (LMM) were used to test the hypotheses. LMMs were fitted using the lme4 package (version 1.1-26) in the R programming environment (version 4.0.4) employing restricted maximum likelihood (REML) to estimate the parameters. Inferential analyses were performed using the lmerTest package (version 3.1-3) using the Satterthwaite’s approximation to estimate denominator degrees of freedom for F statistic and to obtain p-values. In all models, Time (pretest, posttest) was included as a fixed effect and Subject (intercept) as a random effect. An alpha-level of 0.05 was used to determine significance. The specific details of the statistical models are presented in the result section.

## Results

Results are reported in the following order: (1) descriptive results of change in biomarker concentrations at the group level, (2) confirmatory results relating to the hypotheses and (3) selected exploratory analyses of relevance to H6-H8. For the purpose of brevity, some statistics are included in the supplementary materials (SM1–SM14). Including time of blood sampling (08:00, 9:30, 11:00) as a covariate did not change the outcome of any of the confirmatory analyses.

### Descriptive results

The study sample consisted of 50 women and 43 men with an average age of 70.41 years (*SD* = 2.93). A majority (67) had university education, and the remainder high school (16) or elementary school education (9), with education information missing for one participant. 18 participants completed the COG intervention, 26 the PE intervention, 24 the COG + PE intervention, and 25 the PE + COG intervention. All participants were retired and were therefore not working night shifts. One participant had diabetes Type II, which was controlled with Metformin and Novonorm. All participants were non-smokers. Note that the study sample in the present study, which included only those participants who consented to the extended blood analysis, differed slightly from our previous reports of plasma mBDNF concentrations in the original study^25,27^. To allow for a qualitative comparison of acute changes in the three studied biomarkers, based on the same study sample, descriptive results for mBDNF were therefore reported anew.

#### Biomarker concentrations

Median resting concentration at pretest in plasma, in original units, was 144 pg/mL for BDNF (IQR 235, range 28–6820), 86 ng/mL for cortisol (IQR 35, range 23–251) and 424 pg/mL for pro-BDNF (IQR 460, range 39–4937).

**Figure 2:**
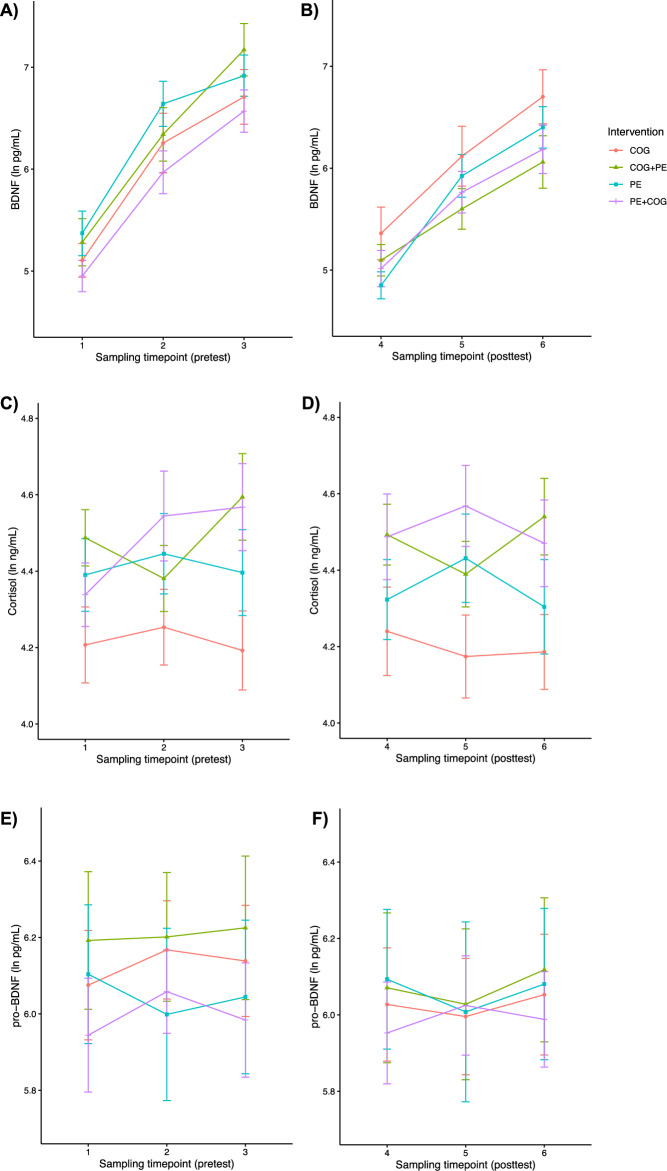
Descriptive results of changes in mBDNF, cortisol and pro-BDNF in response to the acute interventions. Natural log transformed plasma concentrations of mBDNF (**A**,**B**), cortisol (**C**,**D**) and pro-BDNF (**E**,**F**) in response to the acute interventions, at pretest (left column) and posttest (right column). Blood samples were obtained immediately before and after the first intervention, and after the second intervention or rest, at pretest (sampling timepoints 1–3) and at posttest (sampling timepoints 4–6). *COG* cognitive training only, *PE* physical exercise only, *COG + PE* cognitive training and then physical exercise, *PE + COG* physical exercise and then cognitive training. Error bars reflect standard errors.

Transformed concentrations for mBDNF, cortisol and pro-BDNF, at the three sampling timepoints at pretest and posttest revealed different qualitative patterns of change (Fig. 2; for mean concentrations in original units, see SM-1). For mBDNF, there was a significant interaction between Time (pretest, posttest) and Sample (1–3, 4–6), *F*(2) = 6.27, *p* < 0.001, reflecting a steeper increase at pretest than at posttest (for estimated marginal means for significant ANOVA results, see SM-1). The interaction between Intervention (PE + COG, COG + PE, PE, COG) and Time was also significant, *F*(3) = 2.96, p = 0.037, reflecting differential decreases from pretest to posttest in the intervention groups. For cortisol, the interaction between Sample and Intervention was statistically significant, *F*(6) = 2.41, *p* = 0.029, reflecting an apparent pattern of increases in cortisol following physical activity but not following cognitive training. For pro-BDNF, only the main effect of Sample was significant, *F*(2) = 6.18, p < 0.001, reflecting a weakly increasing pattern. As such, mBDNF and pro-BDNF appear to have increased within the session, independent of intervention, whilst cortisol increased primarily following physical activity.

### Confirmatory results

No support was found for H1–H4, and these hypotheses were not followed up in exploratory analyses. For the sake of brevity, these model descriptions and results are therefore presented in the supplementary materials (SM-2, SM-3, SM-4, SM-5). Below we provide model descriptions and confirmatory results for H5-H8.

#### Plasma pro-BDNF and plasma mBDNF are positively associated at rest (Hypothesis 5)

For H5, mBDNF_REST_ acted as dependent variable, and Time and pro-BDNF_REST_ was entered as fixed effects, allowing for interaction with Time, adjusting for adjusting for age, sex and education. The regression coefficient for pro-BDNF_REST_ predicting mBDNF_REST_ was positive and significant (*b* = 0.19, *SE* = 0.09; *ß* = 0.17, *SE* = 0.08), *F*(1, 105.4) = 4.25, *p* = 0.04, supporting the hypothesis. For complete result output see SM-6.

#### Acute changes in plasma cortisol following physical exercise counteract the beneficial effect of temporally coupled changes in plasma mBDNF for cognitive training outcome, when physical exercise precedes but not when it follows cognitive training (Hypothesis 6)

Hypotheses that concerned the influence of exercise-induced changes in biomarker concentrations on cognitive training outcome built directly on previous results (H6-H8). As such, all three models were based on the previously reported model, in which the cognitive composite was the dependent variable, and Time (pretest, posttest), Intervention (COG + PE, PE + COG) and acute change in mBDNF following physical exercise (at pretest) were entered as fixed effects. As such, the fixed effect of Time in this model reflects change in the cognitive composite from pretest to posttest, referred to as cognitive training outcome.

For H6, Cortisol_EX_ was added as a fourth fixed effect, allowing for all interactions. Support for H6 would be derived from a significant four-way interaction, by which less Cortisol_EX_ was related to a stronger association between mBDNF_EX_ and the cognitive composite, when physical exercise preceded but not when it followed cognitive training. In other words, the two intervention groups were expected to differ more in regards to the strength of the association between mBDNF change and cognitive training outcome when Cortisol_EX_ was small compared to large. A significant interaction between Time and Intervention, when mBDNF_EX_ and Cortisol_EX_ are controlled for, would reflect alternative support for the overarching hypothesis that counteracting biomarker effects prevented a beneficial effect of physical exercise preceding cognitive training from emerging at the group level in the original investigation.

The four-way interaction between mBDNF_EX,_ Cortisol_EX_, Time and Intervention (COG + PE, PE + COG), in predicting cognitive performance was not significant, *F*(1, 40) = 0.07, *p* = 0.79, providing no support for the hypothesis. The interaction between Time and Intervention was also not significant, *F*(1, 40) = 1.84, *p* = 0.18, providing no alternative support for the hypothesis. The previously reported three-way interaction between mBDNF_EX,_, Time and Intervention^25^, remained significant also when Cortisol_EX_ was included in the model, *F*(1, 40) = 9.07, *p* < 0.01. No other significant interactions were found. For complete result output see SM-7.

#### Acute changes in plasma pro-BDNF following physical exercise counteract the beneficial effect of temporally coupled changes in plasma mBDNF for cognitive training outcome, when physical exercise precedes but not when it follows cognitive training (Hypothesis 7)

**Figure 3:**
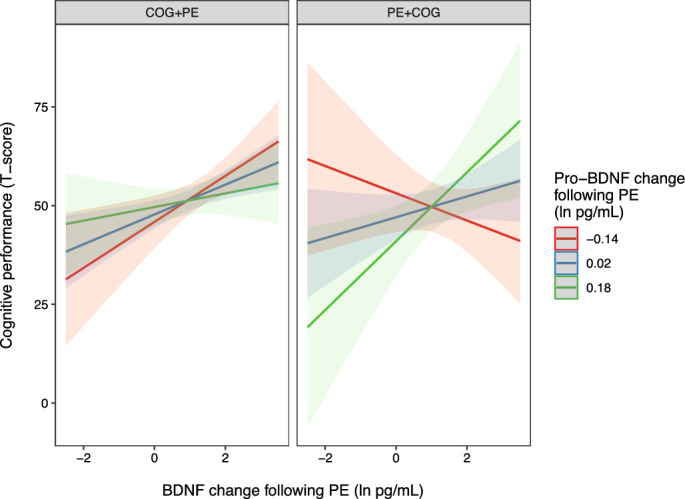
Interaction between changes in mBDNF and pro-BDNF following acute physical exercise, and intervention group, on cognitive performance. Model-implied values illustrating the differential interaction between acute changes in mBDNF and pro-BDNF following physical exercise on cognitive performance (updating working memory, trained tasks with untrained stimuli), collapsed across pre-test and post-test, in the intervention group that received physical exercise before cognitive training (PE + COG) and the group that received the interventions in the reverse order (COG + PE). Shaded areas reflect 95% confidence intervals. Blue reflects the model-implied mean for pro-BDNF change, and red and green reflect one standard deviation below and above the mean, respectively.

The model for H7 and its interpretation was identical to that of H6, except that Pro-BDNF_EX_ was added as a fixed effect together with Time (pretest, posttest), Intervention (COG + PE, PE + COG) and acute change in mBDNF following physical exercise (at pretest). The four-way interaction between pro-BDNF_EX, m_BDNF_EX_, Time and Intervention (COG + PE, PE + COG), in predicting cognitive performance was not significant, *F*(1, 40) = 0.81, *p* = 0.38, providing no support for the hypothesis. The previously reported three-way interaction between mBDNF_EX._ Time and Intervention, remained significant also when pro-BDNF_EX_ was included in the model, *F*(1, 40) = 7.32, *p* = 0.01. An incidental three-way interaction was detected between pro-BDNF_EX, m_BDNF_EX_ and Intervention, *F*(1, 40) = 4.99, *p* = 0.03, reflecting better cognitive performance when greater increases in mBDNF were coupled with greater increases in pro-BDNF following acute exercise, but only when physical exercise preceded cognitive training (Fig. 3). The effect did not differ at pretest and posttest, reflecting that coupling of greater increases of mBDNF and pro-BDNF was for cognitive performance in general and not for cognitive training outcome per se. No other interactions were found, including no significant interaction between Time and Intervention, *F*(1, 40) = 3.06, *p* = 0.09. For complete result output se SM-8.

#### High plasma cortisol levels at rest counteract the beneficial effect of acute changes in plasma mBDNF following physical exercise for cognitive training outcome, when physical exercise precedes but not when it follows cognitive training (Hypothesis 8)

The model for H8 and its interpretation was identical to that of H6 and H7, except that Cortisol_REST_ was added as a fixed effect together with Time (pretest, posttest), Intervention (COG + PE, PE + COG) and acute change in mBDNF following physical exercise (at pretest). The four-way interaction between Cortisol_REST, m_BDNF_EX_, Time and Intervention (COG + PE, PE + COG), in predicting cognitive performance was not significant, *F*(1, 40) = 0.02, *p* = 0.89, providing no support for the hypothesis. No other significant interactions were found. For complete result output see SM-9.

### Exploratory analyses

#### Area under the curve as an alternative measure of acute biomarker change

Since hypotheses 6–8 expanded on previous findings, confirmatory analyses involved the previously used measure of acute change in biomarker concentrations following physical exercise, namely change scores. In a set of exploratory analyses, AUC was used to instead capture biomarker propensity to change across the entire session, as in the confirmatory analyses relating to H1-H5. The aim of these analyses was to explore whether this alternative measure of change may reveal different interactive effects between biomarkers on cognitive training outcome. The same linear mixed effect models were used as for testing hypotheses 6–8, with the exceptions that AUC was used instead of change scores and models were adjusted for age, sex and education.

**Figure 4:**
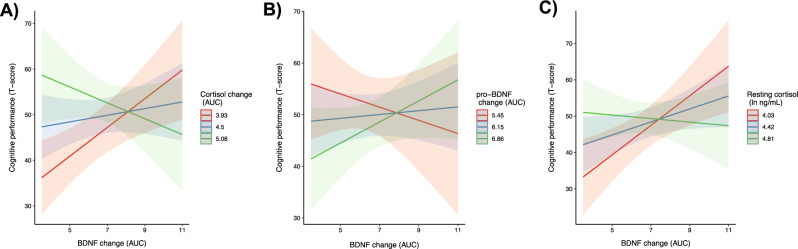
Exploratory biomarkers interactions on cognitive performance. Model-implied values illustrating the significant interaction between acute changes in mBDNF and cortisol (**A**), between acute changes in mBDNF and acute changes in pro-BDNF (**B**), and between acute change in mBDNF and cortisol at rest (**C**), on cognitive performance (updating working memory, trained tasks with untrained stimuli), collapsed across pre-test and post-test. Acute changes are expressed as area under the curve with respect to change (AUC). Shaded areas reflect 95% confidence intervals. Blue reflects the model-implied mean for cortisol/pro-BDNF change, and red and green reflect one standard deviation below and above the mean, respectively.

The four-way interaction between mBDNF_AUC,_ Cortisol_AUC_, Time and Intervention (COG + PE, PE + COG), in predicting cognitive performance was not significant, *F*(1, 39) = 1.37, *p* = 0.25. However, the two-way interaction between mBDNF_AUC_ and Cortisol_AUC_ was significant, *F*(1, 36) = 13.53, *p* < 0.001, reflecting better cognitive performance when greater mBDNF increases were coupled with lesser cortisol increases (Fig. 4A). Furthermore, the two-way interaction between Intervention (COG + PE, PE + COG) and Time (pretest, posttest) was near-significant, *F*(1, 39) = 3.54, *p* = 0.067, but this result likely reflect a regression to the mean rather than greater cognitive improvements in the PE + COG group (SM-10). No other significant interactions were found.

The four-way interaction between mBDNF_AUC,_ pro-BDNF_AUC_, Time and Intervention (COG + PE, PE + COG), in predicting cognitive performance was not significant, *F*(1, 39) = 0.36, *p* = 0.55. However, the two-way interaction between mBDNF_AUC_ and pro-mBDNF_AUC_ was significant, *F*(1, 36) = 1.68, *p* = 0.03, reflecting better cognitive performance when greater mBDNF increases were coupled with greater pro-BDNF increases (Fig. 4B). No other significant interactions were found.

The four-way interaction between mBDNF_AUC,_ Cortisol_REST_, Time and Intervention (COG + PE, PE + COG), in predicting cognitive performance was not significant, *F*(1, 39) = 0.69, *p* = 0.41. However, the two-way interaction between mBDNF_AUC_ and Cortisol_REST_ was significant, *F*(1, 36) = 9.21, *p* = 0.004, reflecting better cognitive performance when greater mBDNF increases were coupled with lower resting cortisol (Fig. 4C). No other significant interactions were found.

The outcome of the exploratory analyses can be summarized as indicating that acute changes in mBDNF across the entire session interacted with parallel changes in cortisol and pro-BDNF, and with cortisol at rest, in their association with the cognitive composite (Fig. 4A–C). As such, a different set of biomarker interactions were indeed detected using area under the curve to capture biomarkers change across the entire session, compared to when using change scores to capture change following physical exercise.

#### Generalizability to other working memory composites

When testing hypothesis 7, an incidental interaction reflected better cognitive performance when greater increases in mBDNF were coupled with greater increases in pro-BDNF following acute exercise, but only when physical exercise preceded cognitive training. This result was found using a cognitive composite that captured performance on working memory tasks that were trained during the intervention period, with the exception that the stimuli was different, as motivated by previous findings on this particular composite^25^. To explore the generalizability of the finding beyond this single composite, analyses were repeated for the two other composites that also targeted the updating working memory: trained working memory task with trained stimuli, and, untrained working memory tasks. The results showed that the three-way interaction was near-significant for trained working memory tasks with trained stimuli, *F*(1,40) = 3.95, *p* = 0.054, and significant for untrained working memory tasks, *F*(1,40) = 4.55, *p* = 0.039, with both interactions following the same pattern of better cognitive performance when greater increases in mBDNF were coupled with greater increases in pro-BDNF following acute exercise, but only when physical exercise preceded cognitive training (SM-11). No other interactions were significant.

The same test of generalizability beyond the single cognitive composite was performed for the exploratory set of AUC analyses reported on in the previous section. The two-way interaction between mBDNF_AUC_ and Cortisol_AUC_ was significant for trained working memory tasks with trained stimuli, *F*(1, 36) = 17.29, *p* < 0.001, and for untrained working memory tasks, *F*(1, 36) = 8.89, *p* = 0.005, with both following the previously detected pattern of better cognitive performance for greater mBDNF increases coupled with lesser cortisol increases (SM-12). The two-way interaction between mBDNF_AUC_ and pro-BDNF_AUC_ was significant for trained working memory tasks with trained stimuli, *F*(1, 36) = 6.25, *p* = 0.017, following the previously detected pattern of better cognitive performance for greater mBDNF increases coupled with greater pro-BDNF increases (SM-13). However, this two-way interaction was not significant for untrained working memory tasks, *F*(1, 36) = 2.00, *p* = 0.166. The two-way interaction between mBDNF_AUC_ and Cortisol_REST_ was significant for trained working memory tasks with trained stimuli, *F*(1, 36) = 6.35, *p* = 0.016, and for untrained working memory tasks, *F*(1, 36) = 6.92, *p* = 0.012, both following the previously detected pattern of reflecting better cognitive performance for greater mBDNF increases coupled with lower resting cortisol (SM-14). No other interactions were significant.

## Discussion

By expanding the biomarker analysis of an already completed randomized-controlled trial in healthy older adult^25^, the present study investigated plasma concentrations of mBDNF, pro-BDNF, and cortisol, at rest and in response to acute interventions, as well as their role in predicting cognitive performance. The results confirmed the hypothesized positive association between mBDNF and pro-BDNF at rest, but did not give support for co-variation among acute changes in mBDNF, pro-BDNF and cortisol. The confirmatory results did also not support the hypothesis that faciliatory effects of mBDNF on cognitive training outcome were counteracted by temporally coupled changes in cortisol or pro-BDNF, or by cortisol at rest. Exploratory results instead revealed greater general cognitive performance when greater acute mBDNF responsiveness was coupled with lesser cortisol responsiveness, greater pro-BDNF responsiveness, and lower cortisol at rest. Below, we discuss the results related to each of the three study aims in more detail.

The first aim was to describe patterns of change in cortisol and pro-BDNF concentrations in blood plasma under conditions of physical exercise and cognitive training. Relative to changes in mBDNF, cortisol and pro-BDNF demonstrated qualitatively different patterns of change at the group level. Whilst mBDNF and pro-BDNF in plasma both displayed gradual acute increases, independent of intervention, such increases appeared less robust for pro-BDNF. In view of previous reports of no change in serum pro-BDNF following acute physical exercise in younger adults (Inoue et al., 2020; Piepmeier et al., 2019), it can be speculated that the acute pro-BDNF response may be different in older adults. Relative to mBDNF and pro-BDNF, which both displayed acute change independent of intervention, cortisol instead appeared to increase primarily following physical exercise, which is consistent with previous reports of increases in plasma cortisol following exercise in younger adults^18^. In contrast, cognitive training was not followed by acute cortisol increases, instead there were indications of decreases, possibly due to recovery from stress induced by the blood sampling. Relative to social psychological stress, which is known to result in a strong cortisol response^29^, cognitive training therefore appears to constitute a relatively mild stressor (or even non-stressor), arguably similar to what has been reported for cognitive testing previously^30^. Importantly, the pattern of cortisol change suggests that the physiological stress was not equivalent in the four interventions, which means that general stress in response to the test situation (e.g. blood sampling) is unlikely to account for the gradual increases observed for mBDNF and pro-BDNF. However, since the study protocol did not include a passive control group (no intervention), we cannot exclude the possibility that the gradual mBDNF and pro-BDNF increases were caused by factors other than the interventions themselves. Taken together, plasma concentrations of mBDNF, pro-BDNF and cortisol displayed qualitatively different patterns of acute change under the same conditions of physical exercise and cognitive engagement, suggesting intervention-general increases in markers of neuroplasticity (mBDNF, pro-BDNF) and exercise-specific increases in markers of physiological stress (cortisol).

The second aim of the investigation was to test a set of hypotheses pertaining to associations between rest levels and acute changes in mBDNF and cortisol, and between mBDNF and pro-BDNF. Consistent with the hypothesis and with previous findings in older adults, mBDNF and pro-BDNF were positively associated at rest^31^. Contrary to the hypotheses, however, no evidence was found for an association between parallel acute changes in mBDNF and cortisol, between changes in mBDNF and cortisol at rest, nor between mBDNF and cortisol at rest. As such, the results are inconsistent with a functional relationship between mBDNF and cortisol, where one inflicts change on the other^8^. Similarly, no evidence was found of an association between acute changes in mBDNF and pro-BDNF, contradicting a functional relationship also between these biomarkers. Thus, whilst acute changes in mBDNF, pro-BDNF and cortisol evidently occur in parallel, they appear to be unfolding independently, suggesting distinct origins.

When interpreting results above, it is important to consider the narrow set of circumstances that the present investigation provided. The acute interventions, comprising aerobic exercise of moderate intensity and/or cognitive training targeting working memory, reflect a very special set of circumstances that may or may not be representative of how the studied biomarkers co-vary under other experimental or more ecological conditions. Another important consideration concerns the limitations in how biomarker change was assessed and measured. Whilst AUC enabled measurement of acute biomarker changes across the entire session, capturing a general propensity for change, information regarding intervention timing was effectively lost. The low resolution at which acute change was measured, with only three timepoints, may also have limited the analytical sensitivity. The study did also not consider non-linear or time-lagged associations, which leaves the possibility for more complex patterns of biomarker co-variation to be discovered in future investigations. Conducting blood sampling in the morning, when for mBDNF and cortisol concentrations are declining, without information of time of awakening, reflect another important limitation.

The third aim of the study was to investigate whether acute changes in mBDNF, cortisol and pro-BDNF interact in their influence on cognitive training outcome. Contrary to the hypotheses, exercise-induced changes in mBDNF did not interact with parallel changes in cortisol or pro-BDNF, or with cortisol at rest, in its association with cognitive training outcome, when cognitive training was preceded by physical exercise. In other words, the beneficial effect of exercise-induced mBDNF for cognitive training outcome, when cognitive training was preceded by physical exercise, did not appear to be counteracted by temporally coupled changes in cortisol or pro-BDNF, or by cortisol at rest. However, an incidental finding revealed better cognitive performance when greater increases in mBDNF were coupled with greater increases in pro-BDNF, and only when physical exercise preceded cognitive training. Exploratory analyses revealed that this finding generalized to two additional cognitive composites also measuring updating working memory ability. In relation to the hypothesis, however, it is important to note that whilst the incidental finding did support an interaction between exercise-induced changes in mBDNF and pro-BDNF on cognitive performance, when physical exercise preceded cognitive training, it did not support a *counteracting* interaction. On the contrary, the nature of the interaction suggests that greater pro-BDNF increases may *boost* the beneficial effect of mBDNF on cognitive performance, which is difficult to reconcile with the role of pro-BDNF in suppressing neuroplastic processes^4,5^. However, since mBDNF is formed from the cleavage of pro-BDNF, greater plasma concentrations of pro-BDNF may simply reflect a larger pool from which mBDNF can be derived^32^. It is therefore conceivable that increases in pro-BDNF, when not overexpressed, can be positive for cognitive performance. However, in this context it should be emphasized that the incidental interaction was associated with cognitive performance *level* and not with performance change from pretest to posttest (i.e., cognitive training outcome), which contradicts a role in facilitating learning and therefore complicates an interpretation involving neuroplastic processes.

Exploratory analyses of relevance to the third study aim revealed indications of a potentially more general role for interactions among mBDNF, cortisol and pro-BDNF, for cognitive performance. In these analyses, AUC was used to capture acute biomarker changes across the entire session, which included both physical exercise and cognitive training, as opposed to change scores capturing acute change following physical activity specifically. As such, AUC reflected a more general measure of biomarker responsiveness to acute interventions, compared to the specific exercise-induced changes captured in the change scores. The AUC results showed that acute changes in mBDNF interacted with parallel changes in cortisol and in pro-BDNF, as well as with cortisol at rest, in its association with cognitive performance. More specifically, greater cognitive performance levels were associated with greater mBDNF increases coupled with lesser cortisol increases, as well as with greater mBDNF increases coupled with lower resting cortisol levels. When testing the generalizability of the finding beyond a single cognitive composite, the same result emerged for two other cognitive composites, also capturing the trained updating construct of working memory. These findings suggest that mBDNF and cortisol responses to acute interventions may indeed have interacting and opposing effects on cognitive performance, which is in line with previous proposals of an integrative system^8^. The exploratory results also showed that greater mBDNF increases coupled with greater pro-BDNF increases were associated with greater cognitive performance level, which is reminiscent of the incidental finding discussed above, and indicates that pro-BDNF and mBDNF may have multiplicable roles in their association with cognitive performance.

As for the incidental findings, however, the biomarker interactions in the exploratory analyses emerged for general cognitive performance level, and not for change in cognitive performance from pretest to posttest. Additionally, the biomarker interactions did not differ depending on whether physical exercise preceded or followed cognitive training, suggesting that their positive associations with cognitive performance were not dependent on a particular timing and therefore may be more trait-like in nature. As such, the exploratory results give indication of an association between a particular combination of biomarker responsiveness and cognitive performance in healthy older adults, but they do not support a mechanism that involve learning via cognitive training.

In summary, the present study provided confirmatory support for a positive association between plasma mBDNF and pro-BDNF at rest, and exploratory support for a trait-like cognitive benefit of exhibiting greater mBDNF responsiveness to acute interventions when coupled with lesser cortisol responsiveness, greater pro-BDNF responsiveness, and lower cortisol at rest, in healthy older adults. Whilst unexpected, the results capture a unique set of findings that should motivate future research to test whether certain profiles of biomarker responsiveness may be associated with more preserved cognition in old age, informing the design of future personalized interventions for cognitive aging.

## Supplementary Information

Supplementary Information.

## Data Availability

Swedish data protection laws prohibit us from putting the data in the public domain, but data and/or analyses can be made available from the corresponding author on reasonable request (Jonna.nilsson@gih.se). For requests that involve specific and well-defined analyses that are in line with the original ethics approval, a data use agreement can be used to effectively transfer the confidentiality obligations of the institution at which the original research was conducted to the institution of the recipient of the data.
