# Supplementary material for: The role of acute changes in mBDNF, cortisol and pro-BDNF in predicting cognitive performance in old age
Source: Sci Rep. 2023 Jun 9;13:9418. doi: 10.1038/s41598-023-35847-5 (PMC10256682; doi:10.1038/s41598-023-35847-5)

The role of acute changes in mBDNF, cortisol and pro-BDNF in predicting cognitive performance in old age

Jonna Nilsson, Maria Ekblom, Marcus Moberg & Martin Lövdén

Supplementary Material 1

Descriptive statistics (means, standard errors) of biomarker concentrations in original units, for all timepoints, separated by intervention group. *COG=cognitive training only, PE=physical exercise only, COG+PE=cognitive training and then physical exercise, PE+COG=physical exercise and then cognitive training.*

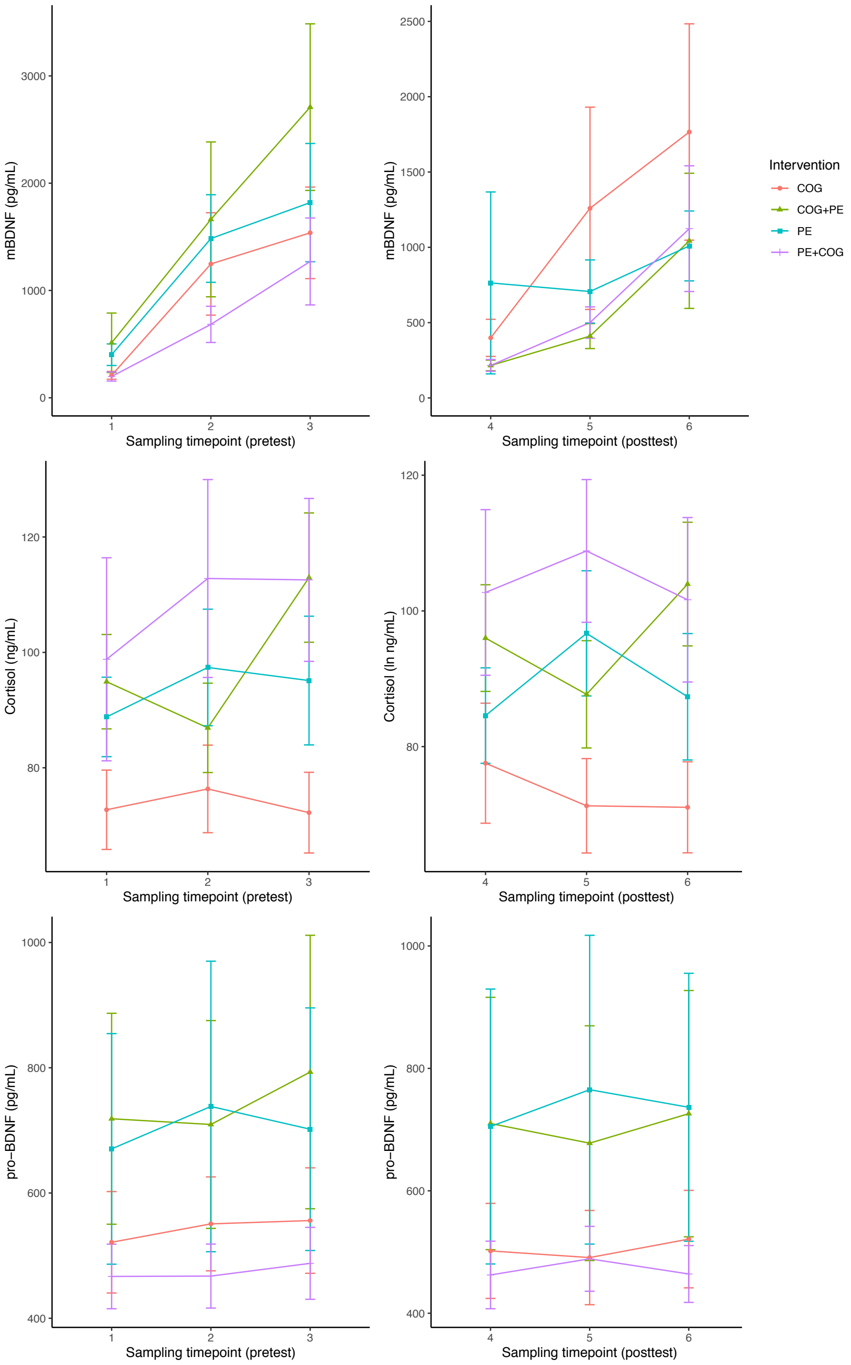

Estimated marginal means (EMM) for significant effects from the repeated-measures ANOVA conducted as part of the descriptive results. Group 1=COG, Group 2=PE, Group 3=COG+PE, Group 4=PE+COG. For complete result output, see below.

mBDNF: Time x Sample interaction, Intervention x Time interaction

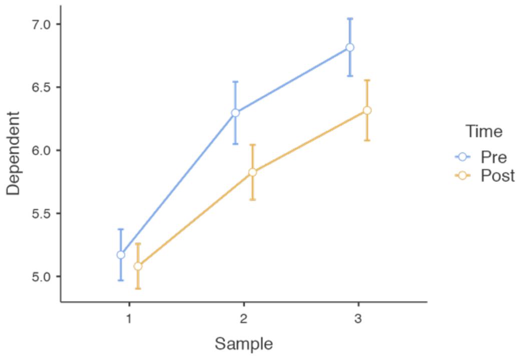

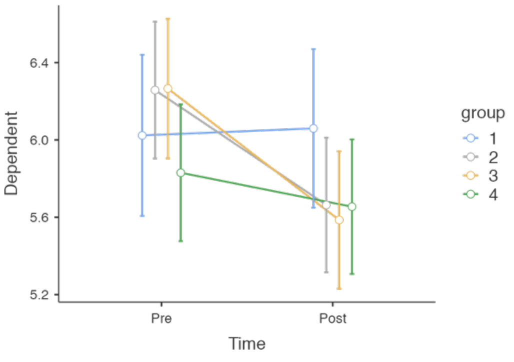

Cortisol: Intervention x Sample interaction

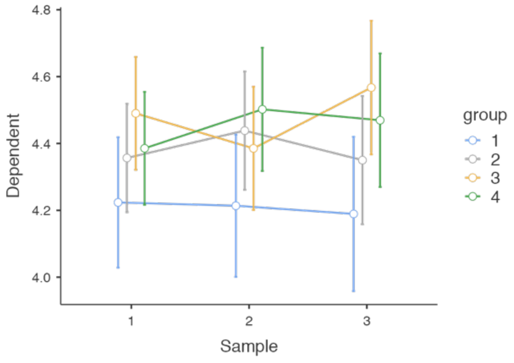

Pro-BDNF: Sample

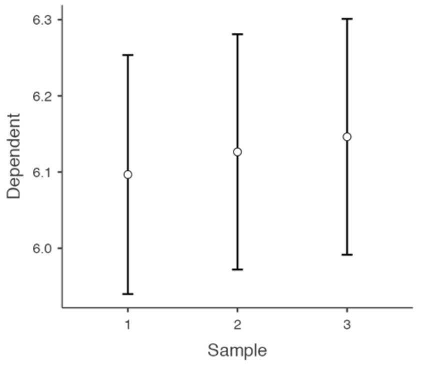

Complete result output for the repeated-measures ANOVA conducted as part of the descriptive results.

Repeated Measures ANOVA: BDNF

Within Subjects Effects

Time	16.912	1	16.9116	13.3922	<.001
Time ∗ group	11.203	3	3.7343	2.9572	0.037
Residual	111.125	88	1.2628		
Sample	192.818	2	96.4088	164.1109	<.001
Sample ∗ group	1.825	6	0.3041	0.5177	0.794
Residual	103.393	176	0.5875		
Time ∗ Sample	4.706	2	2.3530	6.2715	0.002
Time ∗ Sample ∗ group	3.133	6	0.5222	1.3918	0.220
Residual	66.034	176	0.3752		

Note. Type 3 Sums of Squares

[3]

Between Subjects Effects

	Sum of Squares	df	Mean Square	F	p
group	6.499	3	2.166	0.6350	0.594
Residual	300.207	88	3.411		

Note. Type 3 Sums of Squares

Repeated Measures ANOVA: Cortisol

Within Subjects Effects

Time	0.04040	1	0.04040	0.3255	0.570
Time ∗ group	0.11360	3	0.03787	0.3050	0.822
Residual	10.92397	88	0.12414		
Sample	0.08589	2	0.04294	0.4730	0.624
Sample ∗ group	1.31261	6	0.21877	2.4097	0.029
Residual	15.97856	176	0.09079		
Time ∗ Sample	0.13425	2	0.06712	1.6109	0.203
Time ∗ Sample ∗ group	0.18773	6	0.03129	0.7509	0.610
Residual	7.33384	176	0.04167		

Note. Type 3 Sums of Squares

[3]

Between Subjects Effects

	Sum of Squares	df	Mean Square	F	p
group	5.301	3	1.767	1.664	0.181
Residual	93.453	88	1.062		

Note. Type 3 Sums of Squares

Repeated Measures ANOVA: pro-BDNF

Within Subjects Effects

Time	0.42310	1	0.423102	2.8676	0.094
Time ∗ group	0.54501	3	0.181671	1.2313	0.303
Residual	12.68875	86	0.147544		
Sample	0.22030	2	0.110149	6.1810	0.003
Sample ∗ group	0.09517	6	0.015862	0.8901	0.503
Residual	3.06511	172	0.017820		
Time ∗ Sample	0.01553	2	0.007765	0.4663	0.628
Time ∗ Sample ∗ group	0.15841	6	0.026401	1.5855	0.154
Residual	2.86400	172	0.016651		

Note. Type 3 Sums of Squares

[3]

Between Subjects Effects

	Sum of Squares	df	Mean Square	F	p
group	1.828	3	0.6093	0.1897	0.903
Residual	276.168	86	3.2113		

Note. Type 3 Sums of Squares

Supplementary Material 2

Acute changes in plasma cortisol and plasma mBDNF are negatively related, as the interventions unfold (Hypothesis 1).

$mBDNF_{AUC}$ acted as dependent variable and hypothesis testing was performed in two steps. In a first step, Time and $Cortisol_{AUC}$ were added as fixed effect, allowing for interaction with Time, adjusting for age, sex and education. A significant regression coefficient for the predictor of interest was judged as support for the hypothesized association. In a second step, Intervention (COG, PE, COG+PE, PE+COG), entered as dummy variables, were included as fixed effects, allowing interactions amongst all fixed effects. In the case of a non-significant regression coefficient for the predictor of interest in this model, any association detected in the previous step was judged as being dependent on intervention type.

The regression coefficient for $Cortisol_{AUC}$ predicting $mBDNF_{AUC}$ was not significant (b=0.20, SE=0.20; ß=0.08, SE=0.08), F(1, 152.4)=1.01, p=.32, contradicting the hypothesis. When Intervention was added to the model, no interaction was detected between $Cortisol_{AUC}$ and Intervention, F(3, 131.7)=0.95, p=.42, and the regression coefficient for CortisolAUC predicting $mBDNF_{AUC}$ remained non-significant (b=-0.04, SE=0.56; ß=0.02, SE=0.23) , F(1, 136.9)=1.86, p=.18, indicating that the association between $Cortisol_{AUC}$ and $mBDNF_{AUC}$ did not differ by Intervention. An incidental interaction was detected in this model between $Cortisol_{AUC}$ and Time, F(1, 101.5)=4.00, p=.048, reflecting a more negative association between $Cortisol_{AUC}$ and $mBDNF_{AUC}$ at pretest than at posttest. For complete result output and plot of the incidental interaction, see below.

Step 1: Linear mixed effects model with $mBDNF_{AUC}$ as dependent variable, Subject (intercept) as random effect, and Time and $Cortisol_{AUC}$ as fixed effects, allowing for the interaction, adjusting for age, sex and education.

ANOVA results:

	Sum Sq	Mean Sq	NumDF	DenDF	F value	Pr(>F)
**Time**	3,89	3,89	1	109,63	2,80	0,097
**Cortisol AUC**	1,41	1,41	1	152,42	1,01	0,316
**Sex**	5,85	5,85	1	90,39	4,22	0,043
**Age**	0,01	0,01	1	87,77	0,01	0,937
**Education**	4,22	4,22	1	85,00	3,04	0,085
**Time x Cortisol AUC**	1,93	1,93	1	110,33	1,39	0,241

Unstandardized estimates:

	Estimate	Std. Error
**(Intercept)**	0	0
**Time**	-0,82	0,49
**Cortisol AUC**	0,08	0,08
**Female (vs male)**	-0,18	0,09
**Age**	-0,01	0,08
**Education**	-0,15	0,08
**Time x Cortisol AUC**	0,58	0,49

Step 2: Adding Intervention (COG, PE, COG+PE, PE+COG) as a fixed effect to the model in Step 1, allowing for all interactions between fixed effects.

ANOVA results:

	Sum Sq	Mean Sq	NumDF	DenDF	F value	Pr(>F)
**Time**	8,023	8,023	1	101,2	5,7	0,018
**Cortisol AUC**	2,596	2,596	1	136,9	1,9	0,175
**Intervention**	4,225	1,408	3	130,8	1,0	0,392
**Sex**	5,389	5,389	1	87,3	3,9	0,053
**Age**	0,186	0,186	1	84,0	0,1	0,716
**Education**	3,029	3,029	1	83,5	2,2	0,145
**Time x Cortisol AUC ***	5,598	5,598	1	101,5	4,0	0,048
**Time x Intervention**	4,358	1,453	3	101,2	1,0	0,379
**Cortisol AUC x Intervention**	3,981	1,327	3	131,7	0,9	0,419
**Time x Cortisol AUC x Intervention**	4,354	1,451	3	101,7	1,0	0,379

Unstandardized estimates:

	Estimate	Std. Error
**(Intercept)**	9,14	3,74
**Time**	-4,98	2,85
**Cortisol AUC**	-0,04	0,56
**PE (vs COG)**	-0,61	2,77
**COG+PE (vs COG)**	-4,52	3,13
**PE (vs COG)**	-1,53	2,83
**Female (vs male)**	-0,50	0,25
**Age**	-0,02	0,04
**Education**	-0,28	0,19
**Time x Cortisol AUC**	1,14	0,67
**Time x PE (vs COG)**	3,05	3,30
**Time x COG+PE (vs COG)**	1,01	3,67
**Time x PE (vs COG)**	5,20	3,45
**Cortisol AUC x PE (vs COG)**	0,14	0,65
**Cortisol AUC x COG+PE (vs COG)**	0,99	0,72
**Cortsol AUC x PE (vs COG)**	0,27	0,65
**Time x Cortisol AUC x PE (vs COG)**	-0,84	0,77
**Time x Cortisol AUC x COG+PE (vs COG)**	-0,43	0,85
**Time x Cortisol AUC x PE (vs COG)**	-1,26	0,80

Line plot visualizing the Time x Cortisol AUC interaction (marked * in table above):

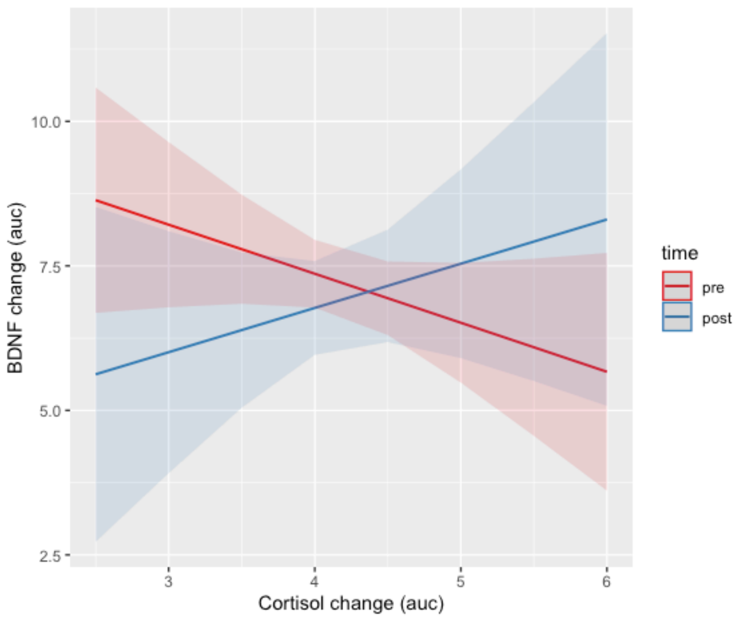

Supplementary Material 3

Plasma cortisol level at rest is negatively related with acute change in plasma mBDNF, as the interventions unfold (Hypothesis 2).

$mBDNF_{AUC}$ acted as dependent variable and hypothesis testing was performed in two steps. In a first step, Time and $Cortisol_{REST}$ were added as fixed effect, allowing for interaction with Time, adjusting for age, sex and education. A significant regression coefficient for the predictor of interest was judged as support for the hypothesized association. In a second step, Intervention (COG, PE, COG+PE, PE+COG), entered as dummy variables, were included as fixed effects, allowing interactions amongst all fixed effects. In the case of a non-significant regression coefficient for the predictor of interest in this model, any association detected in the previous step was judged as being dependent on intervention type.

The regression coefficient for $Cortisol_{REST}$ predicting $mBDNF_{AUC}$ was not significant (b=0.02, SE=0.24; ß=0.01, SE=0.08), $F(1, 139.2)=0.00$, $p=.95$, contradicting the hypothesis. When Intervention was added to the model, an incidental three-way interaction was detected between CortisolREST, Intervention and Time, $F(3, 103.2)=3.27$, $p=.02$, reflecting a more negative association between CortisolREST and $mBDNF_{AUC}$ at pretest than at posttest, but only in the intervention groups that received physical exercise first (PE, PE+COG). The regression coefficient for CortisolREST predicting $mBDNF_{AUC}$ remained non-significant (b=0.00, SE=0.52; ß=0.00, SE=0.17), $F(1, 134.4)=0.07$, $p=.79$, also when Intervention was included in the model. For complete result output and plot of the incidental interaction, see below.

Step 1: Linear mixed effects model with $mBDNF_{AUC}$ as dependent variable, Subject (intercept) as random effect, and Time and $Cortisol_{REST}$ as fixed effects, allowing for the interaction, adjusting for age, sex and education.

ANOVA results:

	Sum Sq	Mean Sq	NumDF	DenDF	F value	Pr(>F)
**Time**	3,71	3,71	1	108,01	2,66	0,106
**Cortisol Rest**	0,01	0,01	1	139,21	0,00	0,947
**Sex**	4,11	4,11	1	87,22	2,94	0,090
**Age**	0,00	0,00	1	85,77	0,00	0,975
**Education**	4,44	4,44	1	84,77	3,17	0,078
**Time x Cortisol Rest**	2,10	2,10	1	108,64	1,50	0,223

Unstandardized estimates:

	Estimate	Std. Error
**(Intercept)**	7,70	3,01
**Time**	-2,01	1,24
**Cortisol Rest**	0,02	0,24
**Female (vs male)**	-0,42	0,24
**Age**	0,00	0,04
**Education**	-0,33	0,18
**Time x Cortisol Rest**	0,34	0,28

Step 2: Adding Intervention (COG, PE, COG+PE, PE+COG) as a fixed effect to the model in Step 1, allowing for all interactions between fixed effects.

ANOVA results:

	Sum Sq	Mean Sq	NumDF	DenDF	F value	Pr(>F)
**Time**	11,71	11,71	1	103,82	8,14	0,005
**Cortisol Rest**	0,10	0,10	1	134,37	0,07	0,789
**Intervention**	1,64	0,55	3	123,87	0,38	0,767
**Sex**	2,61	2,61	1	80,40	1,82	0,182
**Age**	0,05	0,05	1	78,97	0,04	0,851
**Education**	3,79	3,79	1	76,51	2,64	0,109
**Time x Cortisol Rest**	9,04	9,04	1	104,95	6,28	0,014
**Time x Intervention**	13,94	4,65	3	102,50	3,23	0,026
**Cortisol Rest x Intervention**	1,33	0,44	3	124,89	0,31	0,819
**Time x Cortisol Rest x Intervention** *	14,12	4,71	3	103,17	3,27	0,024

Unstandardized estimates:

	Estimate	Std. Error
**(Intercept)**	8,31	3,52
**Time**	-8,05	2,92
**Cortisol Rest**	0,00	0,52
**PE (vs COG)**	1,20	2,84
**COG+PE (vs COG)**	-1,37	3,41
**PE (vs COG)**	-1,36	2,96
**Female (vs male)**	-0,32	0,24
**Age**	-0,01	0,04
**Education**	-0,29	0,18
**Time x Cortisol Rest**	1,86	0,69
**Time x PE (vs COG)**	7,66	3,61
**Time x COG+PE (vs COG)**	0,06	4,29
**Time x PE (vs COG)**	9,11	3,79
**Cortisol Rest x PE (vs COG)**	-0,27	0,66
**Cortisol Rest x COG+PE (vs COG)**	0,28	0,77
**Cortsol Rest x PE (vs COG)**	0,25	0,69
**Time x Cortisol Rest x PE (vs COG)**	-1,91	0,84
**Time x Cortisol Rest x COG+PE (vs COG)**	-0,27	0,98
**Time x Cortisol Rest x PE (vs COG)**	-2,18	0,88

Line plots visualizing the Time x Cortisol Rest x Intervention interaction (marked * in table above). The four plots reflect the different Intervention groups (1=COG, 2=PE, 3=COG+PE, 4=PE+COG).

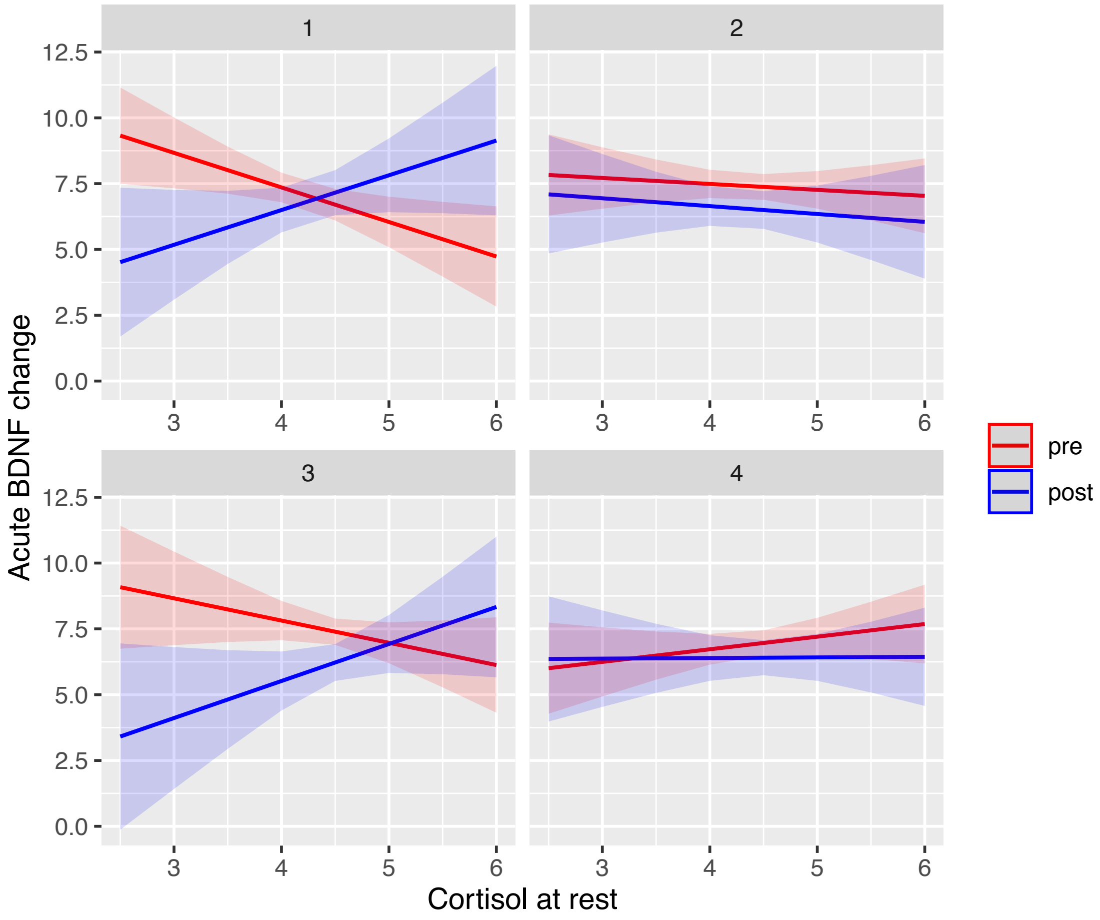

<u>Supplementary Material 4</u>

Plasma cortisol and plasma mBDNF are negatively related at rest (Hypothesis 3).

Linear mixed effects model with $mBDNF_{REST}$ as dependent variable, Subject (intercept) as random effect, and Time and $Cortisol_{REST}$ as fixed effects, allowing for the interaction, adjusting for age, sex and education.

The regression coefficient for $Cortisol_{REST}$ predicting $mBDNF_{REST}$ was not significant (b=0.09, SE=0.16; ß=0.05, SE=0.08), F(1, 141.0)=0.34, p=.56, contradicting the hypothesis. For complete result output see below.

ANOVA results:

	Sum Sq	Mean Sq	NumDF	DenDF	F value	Pr(>F)
**Time**	0,46	0,46	1	108,36	0,79	0,377
**Cortisol Rest**	0,20	0,20	1	141,00	0,34	0,562
**Sex**	3,59	3,59	1	88,20	6,18	0,015
**Age**	0,22	0,22	1	86,73	0,38	0,542
**Education**	1,08	1,08	1	85,74	1,86	0,176
**Time x Cortisol Rest**	0,35	0,35	1	108,97	0,60	0,442

Unstandardized estimates:

	Estimate	Std. Error
**(Intercept)**	4,21	1,97
**Time**	-0,71	0,80
**Cortisol Rest**	0,09	0,16
**Female (vs male)**	-0,40	0,16
**Age**	0,02	0,03
**Education**	-0,16	0,12
**Time x Cortisol Rest**	0,14	0,18

Supplementary Material 5

Acute changes in plasma pro-BDNF and plasma mBDNF are related, as the interventions unfold. (Hypothesis 4).

$mBDNF_{AUC}$ acted as dependent variable and hypothesis testing was performed in two steps. In a first step, Time and pro-$BDNF_{AUC}$ were added as fixed effect, allowing for interaction with Time, adjusting for age, sex and education. A significant regression coefficient for the predictor of interest was judged as support for the hypothesized association. In a second step, Intervention (COG, PE, COG+PE, PE+COG; FE3 below), entered as dummy variables, were included as fixed effects, allowing interactions amongst all fixed effects. In the case of a non-significant regression coefficient for the predictor of interest in this model, any association detected in the previous step was judged as being dependent on intervention type.

The regression coefficient for pro-$BDNF_{AUC}$ predicting $mBDNF_{AUC}$ was not significant (b=0.01, SE=0.14; ß=0.01, SE=0.08), F(1, 112.6)=0.00, p=.94, contradicting the hypothesis. When Intervention was added to the model, no interaction was detected between pro-$BDNF_{AUC}$ and Intervention, F(3, 106.6)=0.68, p=.57, and the regression coefficient for pro-$BDNF_{AUC}$ predicting $mBDNF_{AUC}$ remained non-significant (b=0.10, SE=0.45; ß=0.06, SE=0.25) , F(1, 106.2)=0.26, p=.61. For complete result output see below.

Step 1: Linear mixed effects model with $mBDNF_{AUC}$ as dependent variable, Subject (intercept) as random effect, and Time and pro-$BDNF_{AUC}$ as fixed effects, allowing for the interaction, adjusting for age, sex and education.

ANOVA results:

	Sum Sq	Mean Sq	NumDF	DenDF	F value	Pr(>F)
**Time**	2,26	2,26	1	107,86	1,65	0,202
**Pro-BDNF AUC**	0,01	0,01	1	112,55	0,00	0,944
**Sex**	4,25	4,25	1	85,62	3,11	0,081
**Age**	0,06	0,06	1	84,96	0,05	0,830
**Education**	5,15	5,15	1	85,67	3,77	0,056
**Time x Pro-BDNF AUC**	0,85	0,85	1	106,94	0,62	0,431

Unstandardized estimates:

	Estimate	Std. Error
**(Intercept)**	7,31	3,21
**Time**	-1,26	0,98
**Pro-BDNF AUC**	0,01	0,14
**Female (vs male)**	-0,43	0,25
**Age**	0,01	0,04
**Education**	-0,37	0,19
**Time x Pro-BDNF AUC**	0,13	0,16

Step 2: Adding Intervention (COG, PE, COG+PE, PE+COG) as a fixed effect to the model in Step 1, allowing for all interactions between fixed effects.

ANOVA results:

	Sum Sq	Mean Sq	NumDF	DenDF	F value	Pr(>F)
**Time**	0,56	0,56	1	97,41	0,42	0,519
**Pro-BDNF AUC**	0,35	0,35	1	106,17	0,26	0,611
**Intervention**	3,10	1,03	3	107,11	0,77	0,514
**Sex**	4,60	4,60	1	80,30	3,42	0,068
**Age**	0,02	0,02	1	80,21	0,02	0,894
**Education**	5,23	5,23	1	80,30	3,89	0,052
**Time x Pro-BDNF AUC**	0,07	0,07	1	96,59	0,06	0,814
**Time x Intervention**	4,41	1,47	3	97,59	1,09	0,356
**Pro-BDNF AUC x Intervention**	2,75	0,92	3	106,61	0,68	0,565
**Time x Pro-BDNF AUC x Intervention**	3,77	1,26	3	96,62	0,94	0,426

Unstandardized estimates:

	Estimate	Std. Error
**(Intercept)**	7,11	4,27
**Time**	3,27	2,90
**Pro-BDNF AUC**	0,10	0,45
**PE (vs COG)**	1,12	3,07
**COG+PE (vs COG)**	1,60	3,18
**PE (vs COG)**	-2,56	3,68
**Female (vs male)**	-0,48	0,26
**Age**	0,01	0,04
**Education**	-0,39	0,20
**Time x Pro-BDNF AUC**	-0,56	0,47
**Time x PE (vs COG)**	-4,36	3,29
**Time x COG+PE (vs COG)**	-5,84	3,36
**Time x PE (vs COG)**	-5,84	3,97
**Pro-BDNF AUC x PE (vs COG)**	-0,16	0,50
**Pro-BDNF AUC x COG+PE (vs COG)**	-0,27	0,52
**Pro-BDNF AUC x PE (vs COG)**	0,38	0,60
**Time x Pro-BDNF AUC x PE (vs COG)**	0,65	0,53
**Time x Pro-BDNF AUC x COG+PE (vs COG)**	0,85	0,54
**Time x Pro-BDNF AUC x PE (vs COG)**	0,94	0,65

Supplementary Material 6

Plasma pro-BDNF and plasma mBDNF are negatively related at rest (Hypothesis 3).

Linear mixed effects model with $mBDNF_{REST}$ as dependent variable, Subject (intercept) as random effect, and Time and $pro\text{-}BDNF_{REST}$ as fixed effects, allowing for the interaction, adjusting for age, sex and education.

ANOVA results:

	Sum Sq	Mean Sq	NumDF	DenDF	F value	Pr(>F)
**Time**	0,03	0,03	1	96,80	0,06	0,807
**Pro-BDNF Rest**	2,34	2,34	1	105,42	4,25	0,042
**Sex**	3,56	3,56	1	86,16	6,47	0,013
**Age**	0,25	0,25	1	86,34	0,45	0,505
**Education**	1,56	1,56	1	87,14	2,84	0,095
**Time x Pro-BDNF Rest**	0,07	0,07	1	96,49	0,13	0,721

Unstandardized estimates:

	Estimate	Std. Error
**(Intercept)**	3,44	2,03
**Time**	0,15	0,61
**Pro-BDNF Rest**	0,19	0,09
**Female (vs male)**	-0,40	0,16
**Age**	0,02	0,03
**Education**	-0,21	0,12
**Time x Pro-BDNF**	-0,04	0,10

Supplementary Material 7

Acute changes in plasma cortisol following physical exercise counteract the beneficial effect of temporally coupled changes in plasma mBDNF for cognitive training outcome, when physical exercise precedes but not when it follows cognitive training (Hypothesis 6).

Linear mixed effects model with cognition as dependent variable, Subject (intercept) as random effect, and $Cortisol_{EX}$, $mBDNF_{Ex}$, Time and Intervention (PE+COG, COG+PE) as fixed effects, allowing for all interactions.

ANOVA results:

	Sum Sq	Mean Sq	NumDF	DenDF	F value	Pr(>F)
**Time**	837,80	837,80	1	40	62,86	0,000
**mBDNF**	50,12	50,12	1	40	3,76	0,060
**$Cortisol_{EX}$**	9,23	9,23	1	40	0,69	0,410
**Intervention**	16,71	16,71	1	40	1,25	0,269
**Time x $mBDNF_{EX}$**	33,47	33,47	1	40	2,51	0,121
**Time x $Cortisol_{EX}$**	14,21	14,21	1	40	1,07	0,308
**$mBDNF_{EX}$ x $Cotisol_{EX}$**	3,45	3,45	1	40	0,26	0,614
**Time x Intervention**	24,50	24,50	1	40	1,84	0,183
**$mBDNF_{EX}$ x Intervention**	9,70	9,70	1	40	0,73	0,399
**$Cortisol_{EX}$ x Intervention**	0,00	0,00	1	40	0,00	0,993
**Time x $mBDNF_{EX}$ x $Cortisol_{EX}$**	2,67	2,67	1	40	0,20	0,657
**Time x $mBDNF_{EX}$ x Intervention**	120,89	120,89	1	40	9,07	0,004
**Time x $Cortisol_{EX}$ x Intervention**	0,00	0,00	1	40	0,00	0,995
**$mBDNF_{EX}$ x $Cotisol_{EX}$ x Intervention**	1,99	1,99	1	40	0,15	0,701
**Time x $mBDNF_{EX}$ x $Cortisol_{EX}$ x Intervention**	0,95	0,95	1	40	0,07	0,791

Unstandardized estimates:

	Estimate	Std. Error
**(Intercept)**	52,57	1,85
**Time**	5,94	0,75
**$mBDNF_{EX}$**	2,85	1,47
**$Cortisol_{EX}$**	-5,81	6,98
**Intervention**	-2,93	2,61
**Time x $mBDNF_{EX}$**	0,95	0,60
**Time x $Cortisol_{EX}$**	-2,92	2,83
**mBDNF x $Cortisol_{EX}$**	2,06	4,04
**Time x Intervention**	-1,44	1,06
**$mBDNF_{EX}$ x Intervention**	1,78	2,08
**$Cortisol_{EX}$ x Intervention**	-0,09	9,87
**Time x $mBDNF_{EX}$ x $Cortisol_{EX}$**	0,73	1,64
**Time x $mBDNF_{EX}$ x Intervention**	2,54	0,84
**Time x $Cortisol_{EX}$ x Intervention**	0,03	4,00
**$mBDNF_{EX}$ x $Cortisol_{EX}$ x Intervention**	-2,21	5,72
**$mBDNF_{EX}$ x $Cortisol_{EX}$ x Intervention x Time**	-0,62	2,32

Supplementary Material 8

Acute changes in plasma proBDNF following physical exercise counteract the beneficial effect of temporally coupled changes in plasma mBDNF for cognitive training outcome, when physical exercise precedes but not when it follows cognitive training (Hypothesis 7).

Linear mixed effects model with cognition as dependent variable, Subject (intercept) as random effect, and pro-$BDNF_{EX}$, $mBDNF_{Ex}$, Time and Intervention (PE+COG, COG+PE) as fixed effects, allowing for all interactions.

ANOVA results:

	Sum Sq	Mean Sq	NumDF	DenDF	F value	Pr(>F)
**Time**	515,30	515,30	1	40	31,00	0,000
**mBDNF**	126,42	126,42	1	40	7,60	0,009
**Pro-$BDNF_{EX}$**	5,85	5,85	1	40	0,35	0,556
**Intervention**	4,37	4,37	1	40	0,26	0,611
**Time x $mBDNF_{EX}$**	45,01	45,01	1	40	2,71	0,108
**Time x Pro-$BDNF_{EX}$**	14,20	14,20	1	40	0,85	0,361
**$mBDNF_{EX}$ x Pro-$BDNF_{EX}$**	29,35	29,35	1	40	1,77	0,192
**Time x Intervention**	50,95	50,95	1	40	3,06	0,088
**$mBDNF_{EX}$ x Intervention**	0,17	0,17	1	40	0,01	0,920
**Cortisol x Intervention**	30,68	30,68	1	40	1,85	0,182
**Time x $mBDNF_{EX}$ x Pro-$BDNF_{EX}$**	0,75	0,75	1	40	0,05	0,833
**Time x $mBDNF_{EX}$ x Intervention**	121,77	121,77	1	40	7,32	0,010
**Time x Pro-$BDNF_{EX}$ x Intervention**	18,40	18,40	1	40	1,11	0,299
**$mBDNF_{EX}$ x Pro-$BDNF_{EX}$ x Intervention**	83,01	83,01	1	40	4,99	0,031
**Time x $mBDNF_{EX}$ x Pro-$BDNF_{EX}$ x Intervention**	13,39	13,39	1	40	0,81	0,375

Unstandardized estimates:

	Estimate	Std. Error
**(Intercept)**	51,26	1,92
**Time**	5,06	0,91
**$mBDNF_{EX}$**	3,69	1,34
**$Pro\text{-}BDNF_{EX}$**	-8,61	14,52
**Intervention**	-1,39	2,71
**Time x $mBDNF_{EX}$**	1,04	0,63
**Time x $Pro\text{-}BDNF_{EX}$**	6,36	6,88
**$mBDNF_{EX}$ x $Pro\text{-}BDNF_{EX}$**	13,32	10,02
**Time x Intervention**	-2,25	1,29
**$mBDNF_{EX}$ x Intervention**	0,19	1,89
**$Pro\text{-}BDNF_{EX}$ x Intervention**	-27,89	20,53
**Time x $mBDNF_{EX}$ x $Pro\text{-}BDNF_{EX}$**	1,01	4,75
**Time x $mBDNF_{EX}$ x Intervention**	2,43	0,90
**Time x $Pro\text{-}BDNF_{EX}$ x Intervention**	10,24	9,73
**$mBDNF_{EX}$ x $Pro\text{-}BDNF_{EX}$ x Intervention**	31,67	14,17
**$mBDNF_{EX}$ x $Pro\text{-}BDNF_{EX}$ x Intervention x Time**	-6,03	6,72

Supplementary Material 9

High cortisol levels at rest counteract the beneficial effect of temporally coupled changes in plasma mBDNF for cognitive training outcome, when physical exercise precedes but not when it follows cognitive training (Hypothesis 8).

Linear mixed effects model with cognition as dependent variable, Subject (intercept) as random effect, and $Cortisol_{REST}$, $mBDNF_{Ex}$, Time and Intervention (PE+COG, COG+PE) as fixed effects, allowing for all interactions.

ANOVA results:

	Sum Sq	Mean Sq	NumDF	DenDF	F value	Pr(>F)
**Time**	20,28	20,28	1	40	1,47	0,232
**$mBDNF_{EX}$**	23,87	23,87	1	40	1,74	0,195
**$Cortisol_{REST}$**	0,00	0,00	1	40	0,00	0,990
**Intervention**	1,80	1,80	1	40	0,13	0,719
**Time x $mBDNF_{EX}$**	0,02	0,02	1	40	0,00	0,967
**Time x $Cortisol_{REST}$**	6,07	6,07	1	40	0,44	0,510
**$mBDNF_{EX}$ x $Cotisol_{REST}$**	20,36	20,36	1	40	1,48	0,231
**Time x Intervention**	6,33	6,33	1	40	0,46	0,501
**$mBDNF_{EX}$ x Intervention**	6,97	6,97	1	40	0,51	0,481
**$Cortisol_{REST}$ x Intervention**	1,86	1,86	1	40	0,13	0,715
**Time x $mBDNF_{EX}$ x $Cortisol_{REST}$**	0,76	0,76	1	40	0,06	0,815
**Time x $mBDNF_{EX}$ x Intervention**	0,23	0,23	1	40	0,02	0,897
**Time x $Cortisol_{REST}$ x Intervention**	4,60	4,60	1	40	0,33	0,566
**$mBDNF_{EX}$ x $Cotisol_{REST}$ x Intervention**	9,30	9,30	1	40	0,68	0,416
**Time x $mBDNF_{EX}$ x $Cortisol_{REST}$ x Intervention**	0,25	0,25	1	40	0,02	0,894

Unstandardized estimates:

	Estimate	Std. Error
**(Intercept)**	53,07	22,91
**Time**	11,95	9,84
**$mBDNF_{EX}$**	19,72	14,97
**$Cortisol_{REST}$**	0,06	5,14
**Intervention**	-11,73	32,39
**Time x $mBDNF_{EX}$**	-0,27	6,43
**Time x $Cortisol_{REST}$**	-1,47	2,21
**$mBDNF_{EX}$ x $Cortisol_{REST}$**	-4,08	3,35
**Time x Intervention**	-9,44	13,91
**$mBDNF_{EX}$ x Intervention**	15,07	21,16
**$Cortisol_{REST}$ x Intervention**	2,67	7,27
**Time x $mBDNF_{EX}$ x $Cortisol_{REST}$**	0,34	1,44
**Time x $mBDNF_{EX}$ x Intervention**	1,18	9,09
**Time x $Cortisol_{REST}$ x Intervention**	1,81	3,12
**$mBDNF_{EX}$ x $Cortisol_{REST}$ x Intervention**	-3,90	4,74
**$mBDNF_{EX}$ x $Cortisol_{REST}$ x Intervention x Time**	0,27	2,03

Supplementary Material 10

Visualisation of near-significant interaction between Intervention (PE+COG, COG+PE) and Time in the exploratory linear mixed effects model with cognition as dependent variable, Subject (intercept) as random effect, and Cortisol$_{AUC}$, mBDNF$_{AUC}$, Time and Intervention (PE+COG, COG+PE) as fixed effects, allowing for all interactions.

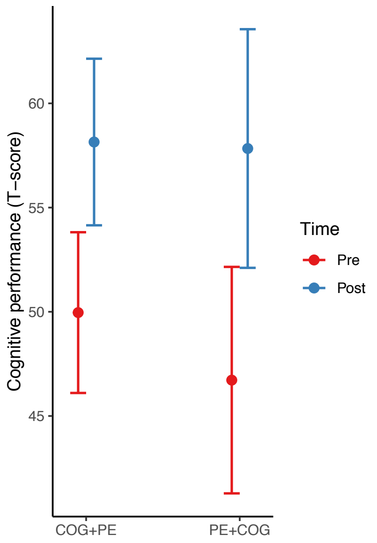

Supplementary Material 11

Line plot visualizing the interaction between pro-BDNF$_{EX}$, mBDNF$_{EX}$ and Intervention on trained working memory tasks with trained stimuli (top panel) and on untrained working memory tasks (bottom panel), discovered in exploratory analyses testing the generalizability beyond a single cognitive composite.

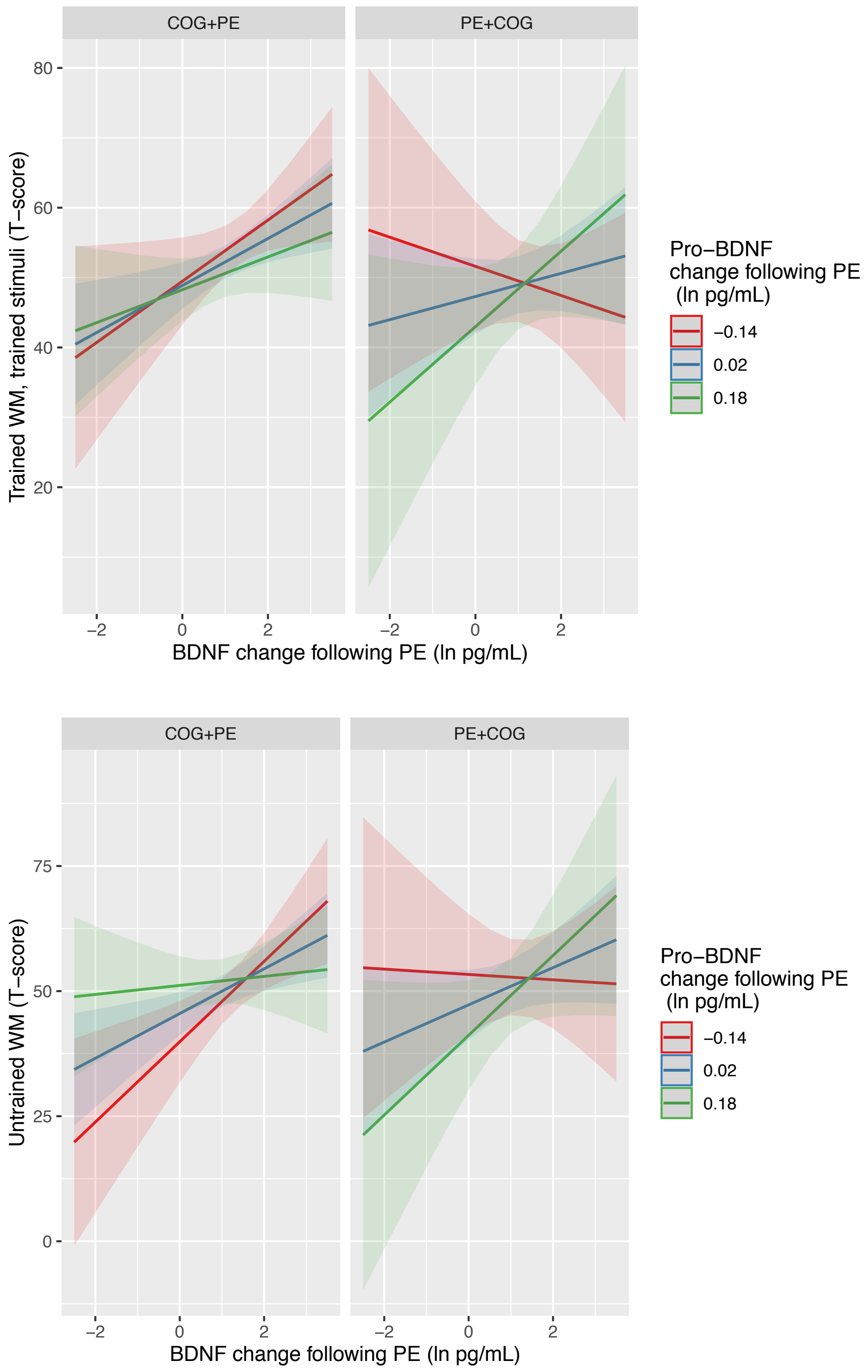

<u>Supplementary Material 12</u>

Line plot visualizing the interaction between $mBDNF_{AUC}$ and $Cortisol_{AUC}$ on trained working memory tasks with trained stimuli (top panel) and on untrained working memory tasks (bottom panel), discovered in exploratory analyses testing generalizability beyond a single cognitive composite.

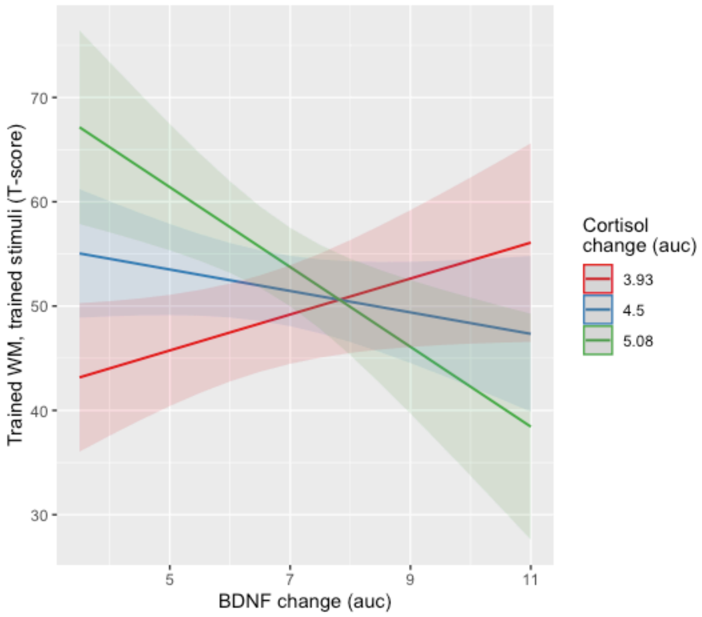

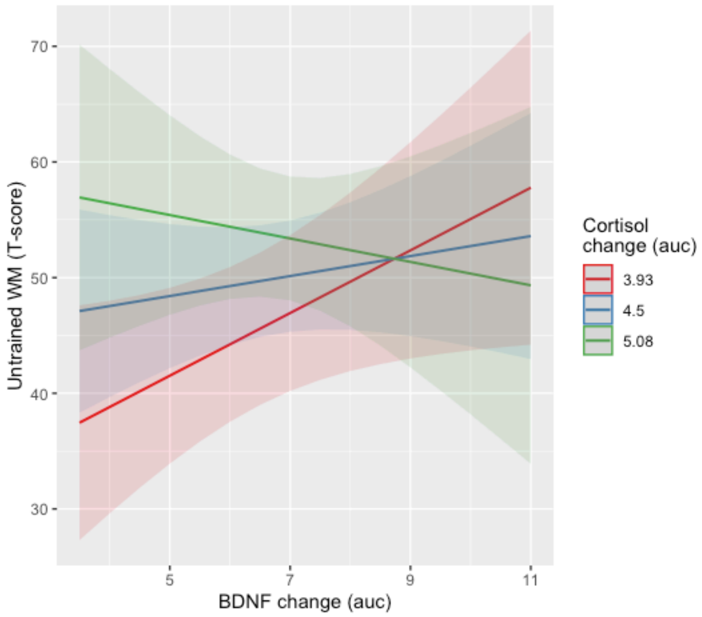

<u>Supplementary Material 13</u>

Line plot visualizing the interaction between $mBDNF_{AUC}$ and pro-$BDNF_{AUC}$ on trained working memory tasks with trained stimuli, discovered in exploratory analyses testing generalizability beyond a single cognitive composite. Note that this interaction was not significant for untrained working memory tasks.

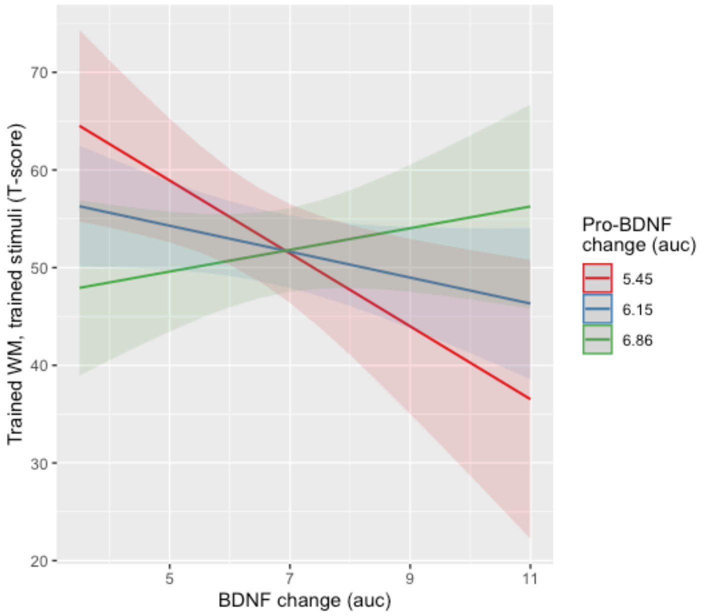

Supplementary Material 14

Line plot visualizing the interaction between $mBDNF_{AUC}$ and $Cortisol_{REST}$ on trained working memory tasks with trained stimuli (top panel) and on untrained working memory tasks (bottom panel), discovered in exploratory analyses testing generalizability beyond a single cognitive composite.

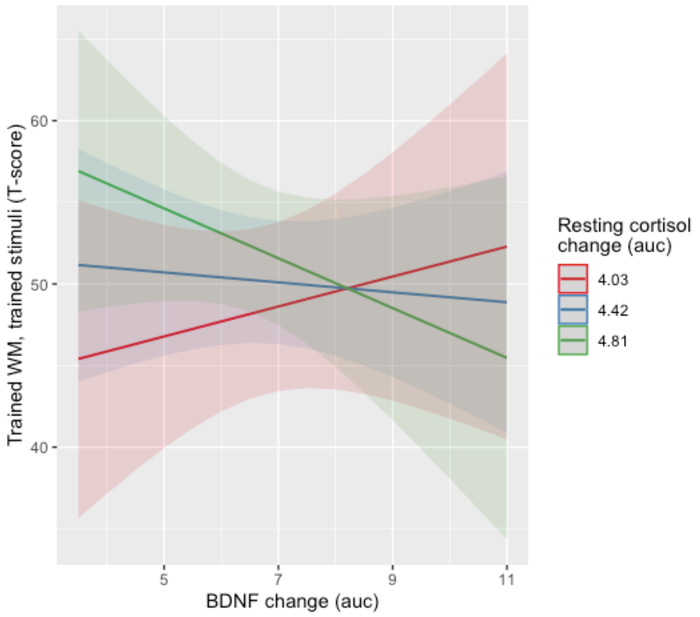

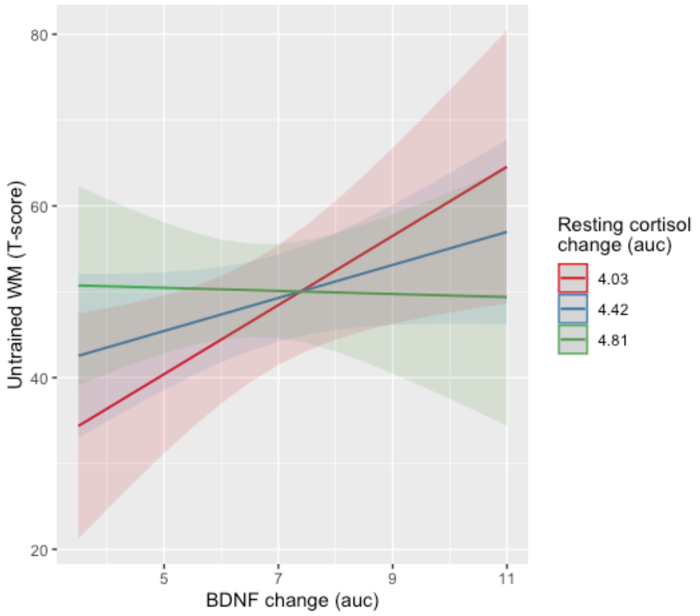

Supplement: Supplementary file 1 — Supplementary Information. [file 41598_2023_35847_MOESM1_ESM.pdf]
